# Vitamin E and Alzheimer’s Disease—Is It Time for Personalized Medicine?

**DOI:** 10.3390/antiox6030045

**Published:** 2017-06-24

**Authors:** Breana Cervantes, Lynn M. Ulatowski

**Affiliations:** Ursuline College, Pepper Pike, OH 44121, USA; breana.cervantes@ursuline.edu

**Keywords:** vitamin E, oxidative stress, Alzheimer’s disease, SNPs, personalized medicine

## Abstract

For the last two decades, it has been hotly debated whether vitamin E—the major lipid-soluble antioxidant, which functions to maintain neurological integrity—is efficacious as a therapy for Alzheimer’s disease. Several factors key to the debate, include (1) which of the eight naturally-occurring vitamin E forms should be used; (2) how combination treatments affect vitamin E efficacy; and (3) safety concerns that most-recently resurfaced after the results of the Selenium and vitamin E Cancer prevention trial SELECT prostate cancer trial. However, with the advent of new genetic technologies and identifications of vitamin E-modulating single nucleotide polymorphisms (SNPs), we propose that clinical trials addressing the question “Is vitamin E an effective treatment for Alzheimer’s disease” should consider a more focused and personalized medicine approach to designing experiments. An individual’s naturally-occurring SNP variants may indeed influence vitamin E’s therapeutic effect on Alzheimer’s disease.

## 1. Vitamin E

For more than 50 years, vitamin E has been recognized as critical for maintaining optimal neurological health. The vital importance of vitamin E is best exemplified by mutations in the tocopherol transfer protein (*TTPA)* gene that—in both animals and humans—manifest as the heritable disorder ataxia with vitamin E deficiency (Ataxia with vitamin E deficiency(AVED); OMIM #277640). *TTPA* encodes the tocopherol transfer protein (TTP), which regulates the whole-body status of vitamin E. AVED is characterized by loss of proprioception, progressive spinocerebellar ataxia, low levels of vitamin E, and elevated oxidative stress [1]. Although there are eight naturally-occurring forms of vitamin E, alpha-tocopherol has garnered the most attention. The focus on alpha-tocopherol is likely because this form has the highest biological activity due to its affinity for TTP [2] and hepatic P450 enzymes preferentially catabolizing non-alpha-tocopherols to water-soluble secreted metabolites [3]. Consequently, plasma and tissues are significantly enriched with the alpha-tocopherol form (~90%) compared to other forms. The overwhelming enrichment of alpha-tocopherol is even more impressive when one considers that Americans consume substantially more non-alpha-tocopherol forms (~75%) as a result of diets high in corn oil [4]. Of course, the discrepancy in consumption and enrichment underscores the body’s sophisticated bioavailability mechanisms [2,3]. Accordingly, the Institute of Medicine’s (IOM) recommended dietary allowance (RDA) is based solely on preventing alpha-tocopherol deficiency, and as a result the term vitamin E is often used interchangeably with the alpha-tocopherol form. However, there is no discrimination with respect to vitamin E uptake—all natural and synthetic E vitamers are absorbed in the small intestines and delivered to chylomicron for liberation into the liver via the low-density lipoprotein (LDL) and scavenger receptor class B type 1(SR-B1) [5]. Thus, vitamin E delivery shares a common route of cellular entry with cholesterol. As such, alpha-tocopherol status is normalized to cholesterol levels, which is reflected in skewed vitamin E values during conditions of fat-malabsorption-related diseases like cholestatic liver disease, cystic fibrosis, and Niemann–Pick type C (NPC) [6,7,8]. NPC is a lysosomal-cholesterol storage disease that shares neurodegenerative characteristics reminiscent of AD and therefore has been referred to as childhood Alzheimer’s disease [9]. 

Functionally, alpha-tocopherol is accepted as the major lipid-soluble antioxidant that maintains cell integrity by preventing lipid peroxidation in cellular membranes [10]. Thus, it is reasonable to hypothesize that oxidative stress-related diseases may benefit from vitamin E supplementation, specifically alpha-tocopherol. The knowledge of increased oxidative stress in human patients suffering from neurological conditions such as AD, Parkinson’s disease, and Down’s syndrome has led to many studies to test the vitamin E efficacy hypothesis [11,12,13,14,15].

## 2. Vitamin E and Neurological Health

Oxidative stress is known to increase with age [16], and as such, humans harbor enzymatic and non-enzymatic compensatory mechanisms—including vitamin E anti-oxidant properties—to combat free radical perturbations. Since vitamin E is an essential micronutrient, humans must consume the vitamin in order to maintain adequate levels. Alarmingly, 90% of the population does not consume the RDA of 15 mg/day but average closer to half that value—around 7 mg/day [17]. The consequences of low vitamin E intake on cognitive decline are exemplified in several studies. In a group of nearly 3000 elderly healthy women who were followed for three years, it was found that individuals who consumed higher vitamin E-containing foods exhibited reduced cognitive decline per an adaptation of the Mini Mental State Examination (MMSE) [18,19]. Similarly, healthy individuals who participated in the Women’s Health Study were shown to have less cognitive decline when consuming higher levels of vitamin E supplementation [20]. Together, these studies support the notion that adequate vitamin E supports neurological health and raises the concern that unrecognized sub-clinical deficiency of vitamin E may contribute to cognitive decline as individuals age. Notably, vitamin E is not a biomolecule that is routinely tested. 

## 3. Alzheimer’s Disease

Alzheimer’s disease (AD) is a devastating progressive neurological condition associated with advanced age. AD is characterized initially by short-term memory loss which progresses to confusion, mood swings, and an inability to perform daily tasks. Currently, AD is estimated to affect about 5 million people in the United States [21]. As a result of our aging baby boomer population, this number is predicted to triple within the next 30 years [21]. The financial burden is staggering, conservatively estimated at over 200 billion dollars annually [22]. Thus, there is great need to develop therapies to combat AD. Pathologically, AD is characterized by accumulation of hyperphosphorylated tau protein and the formation of amyloid-βeta (Aβ) senile plaques [23]. More recently, the contribution of oxidative stress has become increasingly recognized and investigated in the context of AD etiology [24,25].

## 4. Vitamin E and Alzheimer’s Disease

A concrete connection between vitamin E and AD is the significant decrease of vitamin E in the cerebrospinal fluid (CSF) and plasma of AD patients [26,27]. Other studies have individually corroborated the results of lower CSF [28] or lower plasma [29,30] vitamin E. However, in smaller studies, there was no difference in vitamin E CSF or plasma levels [31,32]. Overall, given the earlier studies citing the positive associations between vitamin E supplementation and cognitive health [18,20], these observations provide merit and rationale to support direct studies of alpha-tocopherol supplementation as an intervention strategy in AD. Moreover, vitamin E is relatively inexpensive. Importantly, studies analyzing the correlation between plasma and CSF concentrations, as well as the fact that vitamin E is an essential nutrient (i.e., humans do not synthesize the vitamin) provide support for the nutrient’s ability to effectively cross the blood brain barrier [33,34].

Several clinical studies have investigated the efficacy of vitamin E with respect to AD, yielding inconsistent results [11,20,35,36,37,38]. Some of the confounding factors contributing to analyzing vitamin E-related AD studies include the form of vitamin E used, combination studies with other nutrients and pharmaceuticals, study length, and the stage of neurological acuity. 

Regarding different vitamers, at the cellular level, studies have sought to delineate the role of various vitamin E forms in the molecular underpinnings of AD progression, including the classic risk factors of oxidative stress, inflammation, and cholesterol homeostasis [39,40,41]. The physiological consequences of cell culture studies remain a question due to the absence of TTP’s inherent discrimination for non-alpha tocopherol. Interestingly, a recent randomized control study using human samples from the Rush Memory and Aging Project (MAP) determined there were micro-locations of gamma-tocopherol that correlate with amyloid-beta plaques burdens [42]. These results indeed give credence to investigating other vitamin E forms.

Table 1 focuses on a small sampling of the studies that contribute specifically to the alpha-tocopherol and AD conundrum—the emphasis of this paper because alpha-tocopherol is still considered the most bioavailable form of vitamin E [43]. A clinical trial of over 600 patients with mild-to-moderate AD who were taking an acetylcholinesterase inhibitor at the time of the study were assigned to a daily combination of memantine and/or 2000 IU alpha-tocopherol proved to slow cognitive decline based on the AD Cooperative Study/Activities of Daily Living (ADCS-ADL) Inventory [44,45]. Notably, there were no adverse effects of the extreme levels of alpha-tocopherol used in this study, even after the nearly three-year follow-up period. In another randomized control study—also using 2000 IU alpha-tocopherol and/or a combination of the monoxidase amine (MAO) inhibitor selegiline—individuals with moderate AD exhibited differences in primary outcome measures at the conclusion of the two-year study. The primary outcomes included presentation of one of the following: death, institutionalization, inability to perform daily tasks based on the Blessed-Dementia Scale, or severe dementia per a Clinical Dementia Rating of 3. It should be noted that (1) the statistical efficacy of alpha-tocopherol was observable after adjustment for the baseline Mini-Mental State Examination and (2) the placebo group—although randomly assigned—had higher baseline MMSE scores [11]. In a third study that employed 2000 IU alpha-tocopherol in combinations with donepezil—an acetylcholinesterase inhibitor—to treat individuals with a more advanced sub-group of mild cognitive impairment resulted in essentially an unimpressive effect of vitamin E treatment versus placebo [38]. The results from these three studies speak to two points that have elicited debate regarding efficacy of vitamin E as an AD therapeutic: (1) the timing of vitamin E administration and (2) the safety of high-dose alpha-tocopherol. Relative to the first point, the benefit of vitamin E is skewed towards a pre-emptive measure to attenuate cognitive decline. Concerning the latter point, the amounts administered in all studies were five times the doses (400 IU) often cited as the threshold in all-cause mortality meta-analysis [46,47]. Clearly, the safety of high-dose vitamin E treatment has been a hot-bed of controversy, especially since the all-cause mortality study [46] followed by the early termination of SELECT trial—a large prostate cancer clinical trial using a combination of selenium and vitamin E (400 IU) that was cancelled early due to no efficacy in preventing prostate cancers [48]. Interestingly, a meta-analysis that analyzed an unprecedented 57 studies published between 1988 to 2009 determined there was no overall risk of all-cause mortality at any vitamin E dosage [47]. These analyses included more than 250,000 individuals in studies ranging from 28 to nearly 30,000 participants. The safety concerns should obviously remain a real consideration in future study designs; however, it is noteworthy that together these studies found no adverse effects in the specific study populations.

In another study, AD patients that consumed 800 IU alpha-tocopherol daily for six months were ultimately classified as vitamin E responders and non-responders—based on an individual’s glutathione sulfide (GSSG) enzymatic oxidative stress status and cognitive function as assessed by several measures, including the Mini Mental State Examination, Blessed-Dementia Scale, and Clock Drawing Test [19,49,50]. Outcomes of vitamin E responders were significantly more positive than the non-responders, who alarmingly actually fared worse than the placebo control group [37]. The researchers in the responders/non-responders study segregated the individuals based on the trials outcomes, rather than by a molecular fingerprint that would identify individuals prior to the treatments in the study. The results of this study evoked another confounding issue for future vitamin E research. Specifically, how will researchers design clinical trials in order to identify and stratify individuals based on vitamin E responsiveness? Given the historically controversial research regarding the efficacy of vitamin E in the treatment of heart disease and cancer [46,48], understanding the molecular mechanism of vitamin E responders should be a critical focus for future investigations.

## 5. Vitamin E and Personalized Medicine

The question remains, why are the results of the vitamin E and AD studies so variable? It is interesting to speculate that naturally-occurring single nucleotide polymorphisms (SNPs) could play a role in whether an individual is responsive to vitamin E treatment. Certainly, SNPs have been in support of this notion, several genome-wide association studies (GWAS) have revealed an association between gene variants and plasma vitamin E levels and/or bioavailability [52,53,54]. Given the relationship between vitamin E and cognitive health outlined above [20,35], one would hypothesize that individuals with genetic profiles contributing to low plasma alpha-tocopherol levels may be more amenable to vitamin E therapy. Furthermore, the notion that oxidative stress is a risk factor of AD; and by virtue of vitamin E acting as the major lipid-soluble antioxidant, individuals with an inherently low plasma alpha-tocopherol level may be more responsive to vitamin E therapy to combat oxidative stress-associated AD. At this time, there are no identified SNPs that connect vitamin E to AD risk. However, theoretically, any SNPs in genes involved in the absorption (SR-B1, NPC1L1, CD36) uptake (APOB, APOE, LPL, SR-B1), hepatic transport (TTPA), or egress (ABCA1, ABCG1) of alpha-tocopherol level modulation are candidates for affecting the therapeutic response of vitamin E for AD treatment. Notably, a SNP at −980 in the *TTPA* promoter region was shown to influence vitamin E plasma levels in humans [52]. Moreover, other studies have shown connections between vitamin E and oxidative stress-related genes like myeloperoxidase [55], speaking to the connection between vitamin E, oxidative stress, and AD. Table 2 summarizes the GWAS-identified SNPs that affect vitamin E levels or mechanism.

Together, these studies provide a compelling rationale to investigate if such SNPs may help explain the responders/non-responders AD trial [37], as well as predict an individual’s responsiveness to vitamin E intervention in other clinical trials. At the molecular level, previous work supports the connection. It was reported that multiple naturally-occurring SNPs in the *TTPA* promoter had an effect on transcriptional activity [60]. Collectively, these insights suggest that the effectiveness of vitamin E therapy may relate to the SNPs in vitamin E-related genes. These results give rise to a personalized medicine approach that proposes the use of an individual’s SNP profile as an alternative method to stratify study participants and/or analyze data in clinical trials. This notion is justified given the information made available via the Human Genome Project [61] and the emerging data associating an individual’s genotype and drug responsiveness [62,63,64].

## 6. Conclusions

As the future of vitamin E research progresses, some other considerations merit special attention—namely, the stage of the disease, the dosage and form of vitamin E used, and an individual’s oxidative stress status at baseline. Vitamin E may be an effective agent in pre-emptively slowing the progression of AD, but it is not likely to be efficacious in reversing disease symptoms in advanced phases. The debate regarding safe supplementation dosing of alpha-tocopherol seems to be eternal [46,65]. No AD trials—even at 2000 IU alpha-tocopherol—demonstrated an increased risk of mortality. However, we cannot ignore that there have been several clinical trials that have shown increased all-cause mortality of high-dose vitamin E treatment. Regarding toxicity, it should be noted that there have been no reports of adverse effects of high levels of vitamin E from food products [35]. This observation speaks to our earlier point that a large proportion of individuals may have a sub-clinical deficiency of vitamin E that over time contributes to an increased risk of developing AD. Additionally, this underscores the idea that vitamin E status should be routinely monitored, especially in specific populations.

Finally, in light of the variable conclusions, understanding an individual’s vitamin E and oxidative stress molecular footprints are options that were not feasible and/or considered in earlier clinical trials. Advancements in genetic technologies allow for continued and focused research to stratify and explain the present inconclusive evidence and design future trials addressing vitamin E as an effective therapy in AD treatment. Definitely, the significant amount of positive findings justifies more extensive research in order to find cures to combat this devastating progressive neurological disease that physically and mentally transforms individuals.

## Figures and Tables

**Table 1:** Clinical trials examining vitamin E treatment on Alzheimer’s Disease (AD).

Subjects	Treatment/Duration	Results	Reference
613 Patients with mild to moderate AD	2000 IU/day of alpha-tocopherol; 20 mg/day memantine; 2000 IU/day alpha-tocopherol and 20mg/day memantine; Placebo; Duration: 6 months	Slower cognitive functional decline in alpha-tocopherol group	[51]
341 patients with moderate AD	10 mg/day monoamine oxidase inhibitor; 2000 IU/day alpha-tocopherol; Selegiline and alpha-tocopherol; Placebo; Duration: 2 years	Vitamin E slows functional deterioration in moderate AD patients	[11]
769 subjects–subgroup of the AD Cooperative Study [11]	2000 IU vitamin E (increased from 1000 to 2000 after six weeks); 10 mg donepezil (increased from 5 mg to 10 mg after six weeks); Placebo; Duration: 3 years	Vitamin E treatment did not influence progression of AD	[38]
57 AD patients	800 IU vitamin E for 6 months; Placebo; Duration: 6 months	Post-study segregation: Responders and Non-responders. Responders exhibited lower oxidative stress than non-responders with vitamin E treatment. Cognition decreased in non-responders.	[37]

**Table 2:** Genome-wide association study (GWAS)-determined single nucleotide polymorphisms (SNPs) associated with alpha-tocopherol.

Reference SNP	Gene or Nearest Gene	Outcome	Reference
rs964184	BUD13, ZNF259, APOA5	Increases concentrations of serum alpha-tocopherol	[53]
rs12272004	APOA5	Affects blood alpha-tocopherol status	[56]
rs21088622	CYP4F2	Affects catabolism of vitamin E	[57]
rs11057830	SCARB1	Affects alpha-tocopherol uptake	[57]
rs7834588	NKAIN3	Affects fasting blood alpha-tocopherol status following alpha-tocopherol supplementation	[57]
rs10401969	SUGP1	Affects fasting blood alpha-tocopherol status	[58]
rs58542926	TM6SF2	Affects fasting blood alpha-tocopherol status	[54,58]
rs6994076	TTPA	Affects blood alpha-tocopherol status	[52,59]
rs2333227	Myeloperoxidase	Oxidative stress genotype associated with lower serum alpha-tocopherol	[55]
